# 736. Delays in Malaria Recognition and Door to Anti-malarial Time in a South London Hospital

**DOI:** 10.1093/ofid/ofab466.933

**Published:** 2021-12-04

**Authors:** Nisha Patel, Tomasz Materski, Elisa Gonzalez, Solomon Russom, Gurjinder Sandhu

**Affiliations:** Kings College Hospital, London, UK

## Abstract

**Background:**

The prompt recognition and treatment of Plasmodium falciparum is necessary to prevent death. We reviewed data from a cohort of patients presenting with malaria to Kings College Hospital NHS Trust, London.

**Methods:**

Retrospective review of electronic records and drug charts of patients diagnosed with malaria from Jan 2019- March 2021.

**Results:**

109 cases of malaria were identified representing travellers from 11 Sub-Saharan African countries: Nigeria(38%), Sierra Leone(33%), Ivory Coast(10%). The age range varied from 4 to 76 years with a mean of 44, 66% of the cohort was male. 22 cases occurred during the COVID-19 Pandemic. The commonest symptoms were Fever (97%), Headache (92%) and malaise (72%). P. falciparum was present in 99% cases. A travel history was taken in 94% of cases. Malaria was considered by the first clinician in 82% of cases with the second highest differential being a viral illness. In 6 cases, it took 4 to 11 medical reviews before malaria was considered. 29 patients met the UK criteria for severe malaria. Door to antimalarial time varied from 1 to 128 hours, with a median of 7.4 hours. 46% of the cohort received intravenous Artesunate as their first antimalarial. Extreme delays occurred were clinicians did not consider malaria, patients had negative films or a patient did not declare a travel history when asked. 1 patient died of cerebral malaria with a door to needle time of 2hr 3min. Where a reason for delay is documented, drug availability represented the highest cause with mean delay from prescribing antimalarial to giving antimalarial of 2.7 hours. There was no difference in door to antimalarial administration during the COVID-19 Pandemic, but patients did have a delay in presentation to hospital from onset of symptoms, mean 6.2 days pre-pandemic, 10.5 days during pandemic, this was not statistically significant (P= 0.198). 3 patients presenting during the Pandemic had covid-19 swabs prior to admission and 10 had attended primary care services. Number of days between onset of malaria symptoms and presentation to the Emergency Department

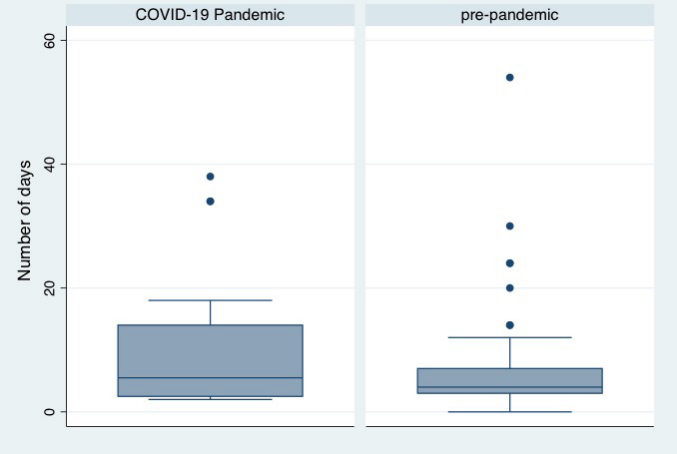

Box plot demonstrating that patients were waiting longer post symptom onset to access care in the Emergency Department. 3 patients had covid swabs in the community and 10 accessed care through their primary care physician.

**Conclusion:**

Our data show that malaria is being considered early in the emergency department however there remain significant delays in administration of treatment. In 6 cases where malaria was not considered early there were delays in diagnosis of up to 5 days. An audit cycle will be completed with the aim of reducing door to antimalarial time.

**Disclosures:**

**All Authors**: No reported disclosures

